# HIF in Gastric Cancer: Regulation and Therapeutic Target

**DOI:** 10.3390/molecules27154893

**Published:** 2022-07-31

**Authors:** Mengqing Li, Guan Li, Xiaodong Yang, Weihua Yin, Guoqing Lv, Shubin Wang

**Affiliations:** 1 Shenzhen Key Laboratory of Gastrointestinal Cancer Translational Research, Department of Oncology, Peking University Shenzhen Hospital, Cancer Institute of Shenzhen-PKU-HKUST Medical Center, Shenzhen 518036, China; limengqing0412@163.com (M.L.); yxdszu@163.com (X.Y.); 2 Department of Pathology, Peking University Shenzhen Hospital, Shenzhen 518036, China; 3 Department of Gastrointestinal Surgery, Peking University Shenzhen Hospital, Shenzhen 518036, China; liguanshenzhen@163.com

**Keywords:** HIF, gastric cancer

## Abstract

HIF means hypoxia-inducible factor gene family, and it could regulate various biological processes, including tumor development. In 2021, the FDA approved the new drug Welireg for targeting HIF-2a, and it is mainly used to treat von Hippel-Lindau syndrome, which demonstrated its good prospects in tumor therapy. As the fourth deadliest cancer worldwide, gastric cancer endangers the health of people all across the world. Currently, there are various treatment methods for patients with gastric cancer, but the five-year survival rate of patients with advanced gastric cancer is still not high. Therefore, here we reviewed the regulatory role and target role of HIF in gastric cancer, and provided some references for the treatment of gastric cancer.

## 1. Introduction

**Figure 1:**
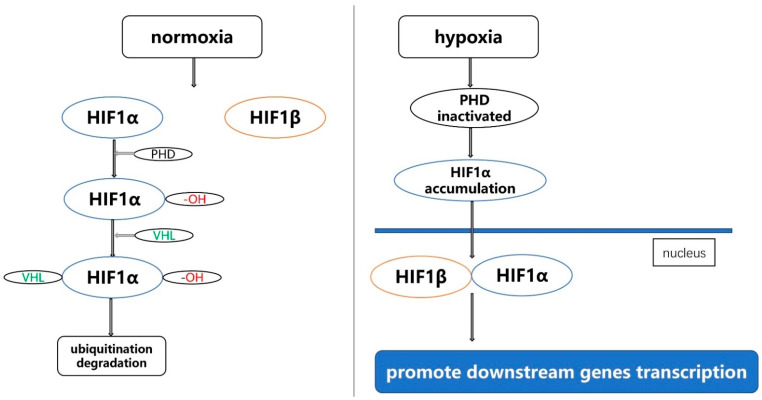
The mechanism of function of HIF1 under normoxia and hypoxia.

The hypoxia-inducible factor (HIF) gene family consists of HIF1, HIF2 and HIF3: HIF1, the most important member of the HIF family, is mainly composed of two subunits, namely HIF1α and HIF1β. When the oxygen concentration is normal, HIF1α is degraded and cannot exist stably. In the case of hypoxia, HIF1α enters the nucleus and combines with HIF1β to promote downstream genes transcription [1]. The specific mechanism is: under normoxia conditions, HIF1α undergoes hydroxylation under the action of prolyl hydroxylase (PHD), and it is recognized and bound by the von Hippel-Lindau tumor suppressor (VHL), when the HIF1α binds to VHL, then it is ubiquitinated and degraded. Under hypoxia, the oxygen-dependent proline hydroxylation reaction is blocked due to the inactivation of PHD, HIF1α is not degraded, and the accumulated HIF1α enters the nucleus and combines with HIF1β to form a dimer, and the dimer regulates the expression of related genes under hypoxic conditions with the participation of transcriptional co-activators such as histone acetyltransferase p300, and finally realizes the adaptation of cells to hypoxia conditions [2,3,4,5,6] (Figure 1). HIF2 is composed of HIF2α and HIF2β. HIF2α is the main functional subunit, and it is rich in tissues such as vascular endothelial cells and fetal lung fibroblasts. After HIF2α is activated, it binds to ARNT to form heterodimerization. Then, it specifically binds to the hypoxia response element of hypoxia-inducible factor (5′-TACGTGCG-3′), thereby upregulating the expression of these genes [7]. It is currently known that HIF3, a less-studied member of the HIF gene family, is composed of HIF3α and HIF3β. The HIF3α gene produces a variety of HIF3α variants, and it is expressed and differentially regulated by hypoxia and other factors. Full-length HIF3α protein functions as an oxygen-regulated transcriptional activator [8]. The HIF gene family plays a very important regulatory role in a variety of diseases, including cancer [9,10,11,12,13,14,15]. For example, Kimberly J Briggs et al. found that HIFα promotes adaptation to hypoxia and stimulates growth in triple-negative breast cancer [16]. Joo-Yun Byun et al. found that HIF-1α promotes cancer stem-like cell phenotype and chemotherapy resistance in head and neck squamous cell carcinoma [17].

**Figure 2:**
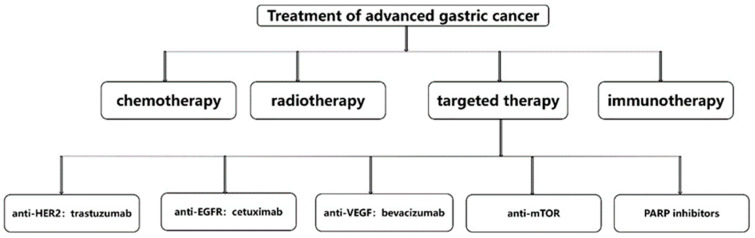
Treatments for advanced gastric cancer.

According to the data of the World Health Organization, the number of new gastric cancer patients in the world in 2020 is 1.09 million, ranking fifth, and the number of gastric cancer deaths in the world in 2020 is 770,000, ranking fourth. Therefore, it can be said that gastric cancer seriously harms the health of people around the world [18]. Current treatments for gastric cancer include systemic chemotherapy, radiotherapy, surgery, immunotherapy and targeted therapy [19]. Even with so many treatments, the median survival for advanced gastric cancer is less than 1 year, and the treatments for advanced gastric cancer include chemotherapy, radiotherapy, immunotherapy and targeted therapy [20]. Targeted therapy for advanced gastric cancer includes anti-HER2, anti-EGFR, anti-VEGF, anti-mTOR, anti-HFG and PARP inhibitors. Representative drugs and specific schematic diagrams are shown in Figure 2 [21]. The above-mentioned targeted therapy drugs have certain curative effects, but the survival period of advanced gastric cancer is always relatively short, so the development of new targeted drugs to prolong the survival period of advanced gastric cancer is a top priority.

In 2016, some scholars found that the antagonist PT2399 targeting HIF2α has good antitumor efficacy in VHL-deficient clear cell renal cell carcinoma [22,23,24]. The discovery of PT2399 suggested that targeting HIF may be a promising target for cancer therapy. Jung-Hyun Park et al. were firstly discover that HIF1α is stably expressed in gastric cancer and may be involved in the progression of gastric cancer [25]. Since then, many studies have proved that HIF plays a regulatory role in the occurrence and development of gastric cancer, and the development of targeted drugs for HIF may be a promising treatment for advanced gastric cancer. Therefore, this review discussed the role of HIF in gastric cancer from its regulation of proliferation, metastasis, apoptosis, drug resistance, angiogenesis, stemness and metabolism of gastric cancer cells, and discussed some HIF-targeted therapies drugs for gastric cancer as well.

## 2. The Regulatory Role of HIF in Gastric Cancer

**Figure 3:**
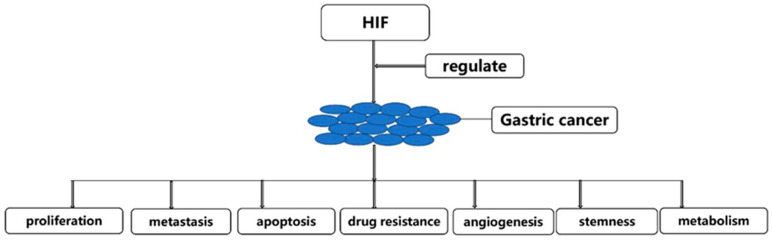
HIF could regulate the occurrence and development of gastric cancer.

From the abstract, we can know that HIF could regulate the occurrence and development of gastric cancer by proliferation, metastasis, apoptosis, drug resistance, angiogenesis, stemness and metabolism of gastric cancer cells (Figure 3). Therefore, we will discuss the progress of HIF regulation of gastric cancer from the above seven aspects.

### 2.1. HIF Regulates Gastric Cancer Progression by Gastric Cancer Cell Proliferation

**Figure 4:**
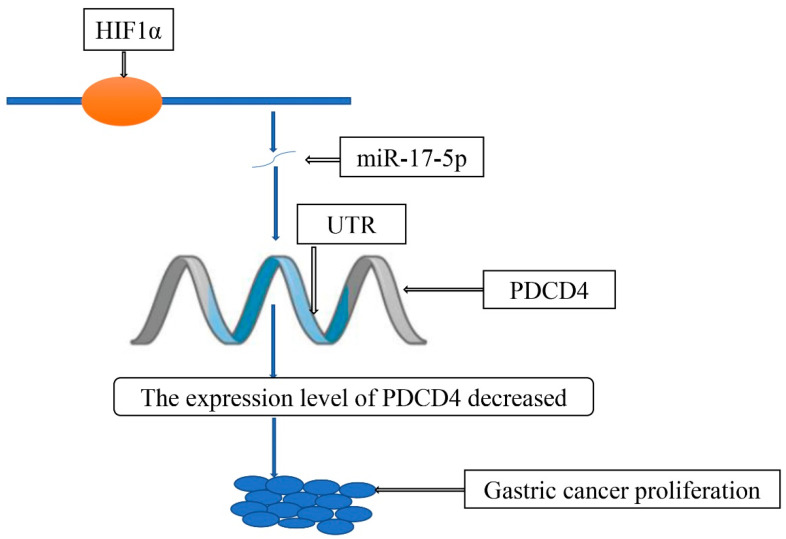
HIF1α/miR-17-5p/PDCD4 axis contributes to the tumor growth of gastric cancer.

We know that tumor cell proliferation plays a huge role in tumor development [26]. HIF also regulates gastric cancer progression by regulating tumor cell proliferation (Table 1). Hai-Yan Piao et al. revealed that HIF1α can bind to the promoter of Hypoxia Yield Proliferation Associated LncRNA (HYPAL) and promote its transcription, which activates the Wnt/β-catenin signaling pathway through HYPAL/miR-431-5p/CDK14 and induces gastric cancer cell proliferation [27]. Jiayu Zhao et al. also discovered that HIF1α could bind to the promoter region of miR-17-5p to activate the transcription of pre-miR-17-5p and miR-17-5p, and miR-17-5p binds to the untranslated region of the gastric cancer suppressor gene programmed cell death 4 (PDCD4), the role of PDCD4 in gastric cancer mainly includes inhibition of cell proliferation, thus leading to the degradation of its mRNA. Finally, HIF1α promotes the proliferation of gastric cancer cells [28] (Figure 4). In addition, according to Lei Hong et al., the overexpression of HIF1α can promote the proliferation of gastric cancer cells, and the tumor suppressor gene Linc-pint can inhibit the proliferation of gastric cancer cells by down-regulating the expression of HIF1α [29].

Many other studies have confirmed that HIF regulates the proliferation of gastric cancer cells [30,31,32,33,34]. Some tumor-targeted drugs such as crizotinib can exert their anti-tumor effects by inhibiting cell proliferation [35]. Maybe, we can find drugs that inhibit the proliferation of gastric cancer cells by targeting HIF in the future, which will help the clinical treatment of gastric cancer.

### 2.2. HIF Regulates Gastric Cancer Progression by Gastric Cancer Cell Metastasis

**Figure 5:**
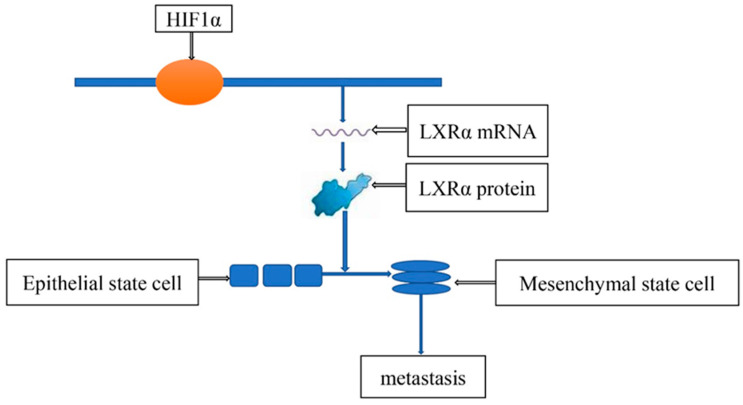
HIF1α-Induced LXRα contributes to the metastasis of gastric cancer cells.

Metastasis causes most cancer deaths [36]. Extensive evidence indicated that HIF plays an important role in gastric cancer metastasis (Table 2). Deng Guan et al. found that HIF1α can promote the epithelial-mesenchymal transition of gastric cancer cells to promote the metastasis of gastric cancer [37]. R Guo et al. proved that HIF1α can directly bind to the promoter of LXRα to promote its transcription, and the increased content of LXRα activates the epithelial–mesenchymal transition of gastric cancer cells, so the metastatic ability of gastric cancer is greatly increased [38] (Figure 5). Xiang Xia et al. revealed that HIF1α could induce gastric cancer cells to release miR-301a-3p-enriched exosomes and promote the metastasis of gastric cancer cells through the MiR-301a-3p/PHD3/HIF-1α positive feedback loop [39]. Furthermore, many studies have found that HIF is involved in the metastasis of gastric cancer cells [40,41,42,43,44,45,46,47,48,49,50,51,52].

Inhibiting tumor metastasis by targeting certain genes is also a major strategy for anti-tumor therapy. For example, Entrectinib inhibits the metastasis of non-small cell lung cancer by targeting ROS proto-oncogene 1(ROS1) and neurotrophic receptor tyrosine kinase (NTRK), ROS1 is a proto-oncogene highly expressed in various tumor cells, and ROS1 protein is a type I integral membrane protein with tyrosine kinase activity, and has achieved satisfactory clinical efficacy [53,54,55,56]. From what we have stated above, we can know that HIF has a certain role in the metastasis of gastric cancer. In the future, it may be a good choice to develop drugs to inhibit the metastasis of gastric cancer by targeting HIF.

### 2.3. HIF Regulates Gastric Cancer Progression by Gastric Cancer Cell Apoptosis

Apoptosis refers to the autonomous and orderly death of cells controlled by genes in order to maintain the stability of the internal environment. It is not a phenomenon of autologous injury under pathological conditions, but a kind of actively striving death process for better adaptation to the living environment. Apoptosis plays a certain role in the occurrence and development of gastric cancer [57] (Table 3). Lili Liu et al. found that HIF1 can promote the expression of the adhesion molecule MGr1-Ag/37LRP by activating ERK and inhibiting the apoptosis of gastric cancer cells [58]. Nadine Rohwer et al. also found that HIF can inhibit the apoptosis of gastric cancer cells by up-regulating alpha5 [59]. However, in fact, the role of HIF in gastric cancer cell apoptosis may be controversial, and some scholars have revealed that HIF can promote gastric cancer cell apoptosis [60,61].

In summary, currently the role of HIF in gastric cancer cell apoptosis is controversial, and more studies are required to clarify the role of HIF in gastric cancer.

### 2.4. HIF Regulates Gastric Cancer Progression by Gastric Cancer Cell Drug Resistance

Drug resistance limits the efficacy of cancer treatment, and addressing drug resistance may be a key issue in cancer treatment [62,63,64,65] (Table 4). Mitsuyoshi Okazaki et al. found that HIF1 promotes drug resistance in gastric cancer cells by affecting the expression of pyruvate kinase muscle 1 (PKM1), PKM1 is a gene associated with chemotherapy resistance in gastric cancer [66]. Based on Yunna Chen et al., using siRNA to knock down HIF1α can reduce the drug resistance of gastric cancer cells and increase the killing effect of 5-fluorouracil on gastric cancer cells [67]. Qun Zhao et al. discovered that HIF1α directly binds miR-27a to promote its expression, and miR-27a promotes drug resistance of gastric cancer cells by inhibiting the expression of MDR1/P-gp, LRP and Bcl-2 [68].

The role of HIF in gastric cancer drug resistance is relatively certain: HIF can promote gastric cancer drug resistance, and many other studies have also confirmed this [69,70,71,72,73,74,75]. HIF promotes drug resistance of gastric cancer cells, and the idea of new drug design revolves around this point.

### 2.5. HIF Regulates Gastric Cancer Progression by Gastric Cancer Cell Angiogenesis

Anti-angiogenesis has always been an important method in designing anti-tumor drugs. For example, bevacizumab can combine with VEGF to inhibit tumor angiogenesis and achieve the effect of inhibiting tumors [76,77]. Zheng Li et al. illustrated that HIF1α can promote angiogenesis in gastric cancer, and this process can be promoted by Natriuretic peptide receptor A (NPRA), NPRA is the most important receptor of atrial natriuretic peptide (ANP), NPRA functions significantly in promoting GC development and progression [78] (Table 5). Ganggang Mu et al. proposed that HIF1α can promote the angiogenesis of gastric cancer by promoting the expression of VEGF-A [79]. E Tang et al. revealed that HIF1α promotes gastric angiogenesis through β-catenin/VEGF signaling, thus promoting gastric cancer progression [80].

Many other studies have confirmed that HIF may be a key factor in gastric cancer angiogenesis [81,82,83,84,85,86]. Inhibition of tumor angiogenesis by developing targeted HIF-related drugs is a promising approach for the clinical treatment of gastric cancer.

### 2.6. HIF Regulates Gastric Cancer Progression by Gastric Cancer Cell Stemness

Cancer stem cells are defined by the American Cancer Society: A cancer stem cell is a small subset of cells present in a tumor that produces heterogeneous tumor cells with the ability to self-renew [87]. Cancer stem cells are considered as important factors in tumor progression [88,89,90,91].

Zhenqin Luo et al. found that HIF1α can promote the progression of gastric cancer by promoting the stemness of gastric cancer cells [92] (Table 6). On the other hand, Zhi-Feng Miao et al. proved that HIF1α promotes peritoneal dissemination by promoting the stemness of gastric cancer cells [93].

At present, there are not many studies on HIF in gastric cancer stem cells, and there is no successful clinical application of drugs targeting cancer stem cells. Therefore, the role of HIF in gastric cancer stem cells needs more research.

### 2.7. HIF Regulates Gastric Cancer Progression by Gastric Cancer Cell Metabolism

Metabolism is closely related to tumors, the metabolism of glucose, lipid and protein in tumors and it is different from normal cells [94,95,96,97,98,99,100,101,102]. Tao Wu et al. discovered that HIF1α promotes gastric cancer progression by promoting glycolysis in gastric cancer cells [103] (Table 7). Xiao-Hong Wang et al. displayed that HIF1α regulates gastric cancer cell glycolysis through the FOXO4/LDHA axis, thereby affecting the progression of gastric cancer cells [104]. According to Jia Liu et al., HIF1α can promote the glycolysis of gastric cancer cells through the circ-MAT2B/miR-515-5p axis, and promote the occurrence and development of gastric cancer cells [105].

Many studies have suggested that HIF1α plays a key role in the metabolism of gastric cancer [106,107,108,109,110].

We know that 5-FU can exert an anti-tumor effect by inhibiting nucleic acid metabolism [111,112]. HIF is closely related to the metabolism of gastric cancer. As a good choice to design drugs for gastric cancer based on this, HIF can promote the progression of gastric cancer by promoting glycolysis under hypoxic conditions.

## 3. Small Molecule Drugs Targeting HIF to Inhibit Gastric Cancer

Small-molecule drugs mainly refer to organic compounds with molecular weights less than 1000, and they have been widely used and mature in theory [113,114,115,116,117]. Apixaban, widely used in clinical practice, is a small molecule drug whose main mechanism is to inhibit the expression of FXa [118,119,120,121,122]. Researchers have discovered many small-molecule drugs that can inhibit gastric cancer progression by targeting HIF (Table 8). Tae Woo Kim et al. found that apigenin, a flavonoid found in traditional medicine, fruits and vegetables, inhibits HIF1α-induced autophagy-related cell death [123]. Noriyuki Egawa et al. demonstrated that low-dose tipifarnib inhibits tumors by inhibiting the expression of HIF1α [124]. Yun-Ning Huang et al. exhibited that dextran sulfate (DS) could inhibit EMT in gastric cancer cells by inhibiting the expression of HIF [125].

There are many small molecule drugs that inhibit the progression of gastric cancer by targeting HIF [74,126,127,128,129,130,131,132,133,134,135,136,137]. Unfortunately, although so many small molecule drugs have been found to inhibit the progression of gastric cancer through HIF, none of them can be used clinically, so more basic and clinical researches are needed.

However, there is a piece of exciting news that the US FDA has approved Merck’s innovative oncology drug Welireg, the first HIF2α inhibitor, for the treatment of VHL syndrome-related tumors. VHL syndrome is a rare and serious genetic disorder associated with a high risk of developing cancer in multiple organs. Prior to Welireg, no systemic therapies were approved for the treatment of VHL-related tumors. Patients suffering from VHL-related tumors treated with Welireg demonstrated high response rates and durable responses [138,139,140,141,142,143,144,145].

Given the success of Welireg, a drug targeting HIF2α in treating VHL syndrome-related tumors, HIF plays a huge role in gastric cancer. Can we look forward to the future that scientists discover that HIF-targeting drugs for the treatment of gastric cancer will benefit patients in the clinic?

## 4. Conclusions

HIF affects the progression of gastric cancer by regulating the proliferation, metastasis, apoptosis, drug resistance, angiogenesis, stemness and metabolism of gastric cancer cells. Many small molecule drugs that inhibit the progression of gastric cancer through HIF have been found in basic experiments, while these drugs have not yet been clinically applied. Given the success of Welireg, a drug targeting HIF2α in treating VHL syndrome-related tumors, HIF plays a huge role in gastric cancer. We look forward to the future where scientists discover that HIF-targeting drugs for the treatment of gastric cancer will benefit patients in the clinic.

## Figures and Tables

**Table 1:** HIF regulates gastric cancer progression by regulating tumor cell proliferation.

Gene	Function	Mechanism	References
HIF1α	promote proliferation	HYPAL/miR-431-5p/CDK14	[27]
HIF1α	promote proliferation	miR-17-5p/PDCD4	[28]
HIF1α	promote proliferation	-	[29]
HIF1α	promote proliferation	-	[30]
HIF1α	promote proliferation	-	[31]
HIF1α	promote proliferation	PI3K/AKT	[32]
HIF1α	promote proliferation	miR-224/RASSF8	[33]
HIF1α	promote proliferation	-	[34]

**Table 2:** HIF regulates gastric cancer progression by gastric cancer cell metastasis.

Gene	Function	Mechanism	References
HIF1α	promote metastasis	EMT	[37]
HIF1α	promote metastasis	LXRα/EMT	[38]
HIF1α	promote metastasis	MiR-301a-3p/PHD3/HIF-1α	[39]
HIF2α	promote metastasis	miR-653-5p/miR-338-3p-NRP1	[40]
HIF1α	promote metastasis	CXCR4	[41]
HIF1α	promote metastasis	PCGEM1/SNAI1	[42]
HIF1α	promote metastasis	P4HB	[43]
HIF1α	promote metastasis	-	[44]
HIF1α	promote metastasis	BC005927/EPHB4	[45]
HIF1α	promote metastasis	GAPLINC	[46]
HIF1α	promote metastasis	Wnt/β-catenin	[47]
HIF1α	promote metastasis	KLF8	[48]
HIF1α	promote metastasis	-	[49]
HIF1α	promote metastasis	RhoE	[50]
HIF1	promote metastasis	67LR	[51]
HIF1α	promote metastasis	-	[52]

**Table 3:** HIF regulates gastric cancer progression by gastric cancer cell apoptosis.

Gene	Function	Mechanism	References
HIF1	inhibit apoptosis	ERK/MGr1-Ag/37LRP	[58]
HIF1α	inhibit apoptosis	alpha5	[59]
HIF1α	inhibit apoptosis	-	[60]
HIF1α	inhibit apoptosis	-	[61]

**Table 4:** HIF regulates gastric cancer progression by gastric cancer cell drug resistance.

Gene	Function	Mechanism	References
HIF1α	promote drug resistance	-	[66]
HIF1α	promote drug resistance	-	[67]
HIF1α	promote drug resistance	miR-27a	[68]
HIF1α	promote drug resistance	-	[69]
HIF1α	promote drug resistance	survivin	[70]
HIF1α	promote drug resistance	p53/NF-kappaB	[71]
HIF1	promote drug resistance	MGr1-Ag/37LRP	[72]
HIF1α	promote drug resistance	-	[73]
HIF1α	promote drug resistance	-	[74]
HIF1α	promote drug resistance	-	[75]

**Table 5:** HIF regulates gastric cancer progression by gastric cancer cell angiogenesis.

Gene	Function	Mechanism	References
HIF1α	promote angiogenesis	-	[78]
HIF1α	promote angiogenesis	-	[79]
HIF1α	promote angiogenesis	β-catenin/VEGF	[80]
HIF1α	promote angiogenesis	VEGF	[81]
HIF1α	promote angiogenesis	VEGF	[82]
HIF1α	promote angiogenesis	miR-382/PTEN/VEGF	[83]
HIF1α	promote angiogenesis	-	[84]
HIF1α	promote angiogenesis	VEGF	[85]
HIF1α	promote angiogenesis	-	[86]

**Table 6:** HIF regulates gastric cancer progression by gastric cancer cell stemness.

Gene	Function	Mechanism	References
HIF1α	promote stemness	-	[92]
HIF1α	promote stemness	-	[93]

**Table 7:** HIF regulates gastric cancer progression by gastric cancer cell metabolism.

Gene	Function	Mechanism	References
HIF1α	promote aerobic glycolysis	-	[103]
HIF1α	promote glycolysis	-	[104]
HIF1α	promote glycolysis	circ-MAT2B/miR-515-5p	[105]
HIF1α	promote glucose Metabolism	-	[106]
HIF1α	promote glycolysis	-	[107]
HIF1α	promote glucose metabolism	-	[108]
HIF1α	promote aerobic glycolysis	-	[109]
HIF1α	promote glucose metabolism	-	[110]

**Table 8:** Small molecule drugs targeting HIF to inhibit gastric cancer.

Drugs	Target	Mechanism	References
apigenin	HIF1α	promote autophagy	[123]
tipifarnib	HIF1α	-	[124]
dextran sulfate	HIF1α	inhibit metastasis	[125]
schisandrin B	HIF1α	inhibit metastasis	[126]
Glaucocalyxin a	HIF1α	inhibit metastasis	[74]
Resveratrol	HIF1α	inhibit metastasis	[127]
Oleanolic acid	HIF1α	inhibit aerobic glycolysis	[128]
ginsenoside Rg3	HIF1α	inhibit angiogenesis	[129]
EGCG	HIF1α	promote apoptosis	[130]
Wogonin	HIF1α	inhibit proliferation	[131]
FS-7	HIF1α	inhibit glycolysis	[132]
TC24	HIF1α	promote apoptosis	[133]
dextran sulphate	HIF1α	inhibit metastasis	[134]
Sulforaphane	HIF1α	inhibit angiogenesis	[135]
Quercetin	HIF1α	promote autophagy	[136]
Celecoxib	HIF1α	promote autophagy	[137]

## Data Availability

Not applicable.
